# Supplementary material for: Impact of spinal fusion on severity health status in scoliotic adolescents with polyhandicap
Source: PLoS One. 2024 Mar 7;19(3):e0300065. doi: 10.1371/journal.pone.0300065 (PMC10919586; doi:10.1371/journal.pone.0300065)
Supplement: Attachment — Submitted filename: review.docx [file pone.0300065.s002.docx]

Thank you for the opportunity to review the article, the authors have tackled an important and difficult topic.

1. The abstract says: We included scoliotic adolescents 37 with polyhandicap who underwent spinal fusion surgeries from 2009 to 2020. Was the surgical technique the same from 2009 to 2020?
2. Hospital stay of 45 days - add important information how were patients treated after surgery? Physiotherapy? Corset treatment? Drug treatment? Other treatment?
3. Did the authors take into account the recommendations of SOSORT?
4. Could the authors elaborate on this sentence?Two independent evaluators performed X-ray measurements to reduce ascertainment bias. In cases of disagreement, the referring surgeon arbitrated the decision.
5. Statistical analysis - the authors did not state to what level of significance they refer their results, moreover, the tables in which there are p-values should also include the level of significance to which the authors refer.
6. Why are descriptive statistics presented as means with standard deviation (SD) for quantitative variables and frequencies for qualitative variables (%)? Why are the median and 1st and 3rd quartile values not presented?
7. Prism GraphPad software - please add more information about the software, there is no reference, this should also be completed.
8. Table 1 - the authors should add the age of the subjects.
9. Results - in addition to tables, the authors should add figures that would enrich the simple statistical methods used.
10. The authors should add what the limitations were in this study.
11. Conclusion - state what practical implications arise from this study, how they can be used - applied.
12. Please add current references 2013-2023 and, please add reference to Prism GraphPad software.
