# Supplementary material for: Impact of spinal fusion on severity health status in scoliotic adolescents with polyhandicap
Source: PLoS One. 2024 Mar 7;19(3):e0300065. doi: 10.1371/journal.pone.0300065 (PMC10919586; doi:10.1371/journal.pone.0300065)
Supplement: Attachment — Submitted filename: Response to Reviewers.docx [file pone.0300065.s003.docx]

**Dear Editor, thank you for all the comments. Here is a detailed point-by-point response corresponding to the suggested changes made to the manuscript:**

**To Academic Editor - March 28th**

1. "Thank you for providing the ethics documentation as reply to our previous request.

Please include an English translation of the ethics committee approval letter as an "Other" file. Please note that no officially translated version of the documents is required, but that the authors themselves can translate the ethics documents for this purpose.

Thank you for your attention. We look forward to hearing from you."

**Authors**

We thank the Academic Editor for this request. We uploaded as required an English translation of the ethics committee approval letter as an “Other” file (Ethics Statement – English version).

2.

2.1) Please provide the specific name of the ethics Committee that approved your study

**Authors**

The Ethics Committee which approved our study is “Terre d’Ethique” Research Committee :

- Institutional Review Board : IORG0007394
- Study label : IRBN492021/CHUSTE (Saint-Etienne University Hospital Centre)

We detailed this information in the Method section (page 6, line 146) and added the IRB proof with this submission (Other “Ethics Statement”)

2.2) In the ethics statement in the Methods, you have specified that verbal consent was obtained. Please provide additional details regarding how this consent was documented and witnessed, and state whether this was approved by the IRB

**Authors**

Thank you for this suggestion. We notice that the written information was submitted and validated to the precited Ethics Committee before caregivers diffusion. We added required precisions on the dedicated paragraph :

“All familial caregivers received written information about the study, according to French law, for retrospective studies. A written consent was collected from all caregivers” in the Methods section (page 6, lines 148-149).

**To Reviewers**

1. The abstract says: We included scoliotic adolescents 27 with polyhandicap who underwent spinal fusion surgeries from 2009 to 2020. Was the surgical technique the same from 2009 to 2020?

**Authors**

To consider this comment we changed the sentence (page 2, line 36) *“We included scoliotic adolescents with polyhandicap who underwent spinal fusion surgeries from 2009 to 2020”* by: “We included between 2009 to 2020, 30 scoliotic adolescents with polyhandicap who underwent spinal fusion performed with the same surgical technique and the same surgeon.”

2. Hospital stay of 45 days - add important information how were patients treated after surgery? Physiotherapy? Corset treatment? Drug treatment? Other treatment?

**Authors**

Each patient received personalized post-operative rehabilitation consisting in medication against spasticity and pain as needed, physiotherapy, and moltened seat confection. We added these notions to the original sentence (page 2, line 47): *The mean hospital length of stay was 45 days.* *During postoperative period, patients received a personalized post-operative rehabilitation procedure with spasticity and pain treatments, physiotherapy, and verticalization (wheelchair sitting and positioning devices such as contoured seat intended to increase postural stability).*

3. Did the authors take into account the recommendations of SOSORT?

**Authors**

We thank the reviewer for this interesting suggestion. Nevertheless, the SOSORT recommendations are for conservative treatment of idiopathic scoliosis. The present study targets non conservative treatment (spinal fusion surgery) for scoliotic adolescents with polyhandicap and could not include these recommendations. Therefore, the present manuscript doesn't add any changes related to the SOSORT recommendations.

4. Could the authors elaborate on this sentence? Two independent evaluators performed X-ray measurements to reduce ascertainment bias. In cases of disagreement, the referring surgeon arbitrated the decision.

**Authors**

Two independent evaluators (HB and EO) performed independent X-ray measurements using the semiautomatic procedures of the Surgimap Spine® software. In cases of disagreement (>6° or >7° differences between two measurements, respectively in frontal and sagittal planes as described by “Wu W, Liang J, Du Y, Tan X, Xiang X, Wang W, et al. Reliability and reproducibility analysis of the Cobb angle and assessing sagittal plane by computer-assisted and manual measurement tools. BMC Musculoskelet Disord. 2014 Dec;15(1):33.”), the referring surgeon (BD) was charged to arbitrate the decision.

To clarify the procedure, we added the following informations (page 7, line 164): “Two independent evaluators (HB and EO) performed X-ray measurements to reduce ascertainment bias. In cases of disagreement (>6° or >7° differences between two measurements, respectively in frontal and sagittal planes), the referring surgeon (BD) arbitrated the decision.”

5. Statistical analysis - the authors did not state to what level of significance they refer their results, moreover, the tables in which there are p-values should also include the level of significance to which the authors refer.

**Authors**

Significance threshold was set at p < 0.05. We added the significance threshold both in the “Statistical analysis” section (page 9, line 211) and below in the Tables (2., 3. and 4.)

6. Why are descriptive statistics presented as means with standard deviation (SD) for quantitative variables and frequencies for qualitative variables (%)? Why are the median and 1st and 3rd quartile values not presented?

**Authors**

We thank the reviewer to offer us the opportunity to clarify our results.

Table 1: we added the mean and median ages at surgery of our subjects (point 8.) and better described our population (number of subjects = n and their related proportions = %)

Table 2: All data presented are quantitative data (clinical scores, body mass index, angles). However, to make it clearer, we made the following corrections, expressing as recommended our data as medians [1^st^ quartile – 3^rd^ quartile]

7. Prism GraphPad software - please add more information about the software, there is no reference, this should also be completed.

**Authors**

We added more accurate information in the method section about the software version we used (page 9, line 211) : Prism GraphPad® software for Windows, version 5.03, San Diego California USA, [www.graphpad.com](http://www.graphpad.com). This software was registered to CHUSE and Saint-Etienne university under the license GPW5-865491-RAG-9037.

8. Table 1 - the authors should add the age of the subjects.

**Authors**

We added the age of the subjects in Table 1(mean/median ages with sd / 1^st^ and 3^rd^ quartile - page 10, line 231)

9. Results - in addition to tables, the authors should add figures that would enrich the simple statistical methods used.

**Authors**

As a way to enrich our statistical methods, we added a study description figure (page7, line 157) as presented above (**Figure 1. Study design**). We pay attention to use Preflight Analysis and Conversion Engine (PACE) digital diagnostic tool before submitting it (file format .tif).

**Fig 1. Study design**. M Months; PSS Polyhandicap Severity Score; mCDS modified Clavien-Dindo-Sink classification; FCA Frontal Cobb Angle; FB Frontal Balance; OBL Pelvic Obliquity; SCA Sagittal Cobb Angle; SB Sagittal Balance; PI Pelvic Incidence; SS Sacral Slope; PT Pelvic Tilt.

We took care to modify the format of the 3 others figures, the numbers in manuscript body, and added respective legends in text conferring to figure caption formatting guidelines.

10. The authors should add what the limitations were in this study.

**Authors**

To consider the reviewer’s comment, we added a « Strengths and Limitations” paragraph at the end of the discussion section paragraph) (page 19, line 396)*. “*Our study demonstrates for the first time, that spinal fusions reduce the global severity of adolescents with polyhandicap. However, it contains several limitations: our study was monocentric and retrospective data collection may have provided methodological bias.”

11. Conclusion - state what practical implications arise from this study, how they can be used - applied.

**Authors**

To highlight the implications for clinical practice we added the sentence (page 20, line 415): “Practitioners should be informed of the importance of performing spinal fusion surgery in early adolescence of PLH patients in order to improve their global health status”.

12. Please add current references 2013-2023 and, please add reference to Prism GraphPad software.

**Authors**

Thank you for this comment. We added Prism GraphPad® software specifications in point 7. (Windows, version 5.03, San Diego California USA, [www.graphpad.com](http://www.graphpad.com)). We have updated the reference 19. Hamouda I, Rousseau MC, Aim MA, Anzola AB, Loundou A, De Villemeur TB, et al. Development and initial validation of the quality of life questionnaire for persons with polyhandicap (PolyQoL). Ann Phys Rehabil Med. févr 2023;66(1):101672. which is the most recent indexed citation dealing with polyhandicap to our knowledge.
