# Supplementary material for: Impact of spinal fusion on severity health status in scoliotic adolescents with polyhandicap
Source: PLoS One. 2024 Mar 7;19(3):e0300065. doi: 10.1371/journal.pone.0300065 (PMC10919586; doi:10.1371/journal.pone.0300065)
Supplement: Attachment — Submitted filename: Response to reviewers - revision.docx [file pone.0300065.s004.docx]

***Dear Editor, Dear Reviewers,***

**We would like to thank you for all the comments, which have helped us to improve the quality of our article. Here are detailed point-to-point responses corresponding to the suggested changes made to our manuscript entitled “Impact of spinal fusion on severity health status in scoliotic adolescents with polyhandicap”. All data underlying the findings of our manuscript is fully available in Supporting Data (dataSPH.xlsx).**

**Reviewer #1**

***Thank you for the submission of your research work to the journal. However, I have few concerns.***

***I do not agree with the authors stating severe scoliosis as Cobb angle more than 50 degrees. Severe scoliosis is usually defined when Cobb angle of more than 80 - 100 degrees.***

- Thank you for your interesting comment. To date and for CP adolescents, recent publications classify the severity of scoliosis in GMFCS III to V individuals as follows : mild scoliosis for a Cobb angle of 10–19°, moderate scoliosis for an angle of 20–39°, and severe scoliosis for an angle of ≥ 40° (1). These values are commonly extrapolated for PIMD/polyhandicap individuals as illustrated by previous published articles (2-4).
- We agree with you, the term “severe scoliosis” may be inappropriate thus we changed in the manuscript the term severe scoliosis to “scoliosis with Cobb angle >50° ” (p5 line 112, p5 line 116, p7 line 154, p23 line 487)
- Cobb angle values are not sufficient to determine the indication for surgery, and we totally agree that spinal fusion surgeries are often carried out at higher Cobb angles, as you describe. We added this sentence to clarify those indications in Material and methods – participants section (p7lines 154-57) “Additionally, surgical indication was determined through a multidisciplinary evaluation, considering various parameters including respiratory and abdominal disorders, complications in wheelchair installation, and severe axial hypotonia.”
- We have paid particular attention to report sagittal and frontal spinal curvature angles (FCA, SCA). As shown in Table 2, our operated patients had preoperative Cobb angles well above 50°.

References:

(1) Vinje S, Terjesen T, Kibsgård T. Scoliosis in children with severe cerebral palsy: a population-based study of 206 children at GMFCS levels III–V. Eur Spine J 2023;32:4030–6. <https://doi.org/10.1007/s00586-023-07868-1>.
(2) Rousseau MC, Mathieu S, Brisse C, Motawaj M, Grimont E, Auquier P, et al. Aetiologies, comorbidities and causes of death in a population of 133 patients with polyhandicaps cared for at specialist rehabilitation centres. Brain Injury 2015;29:837–42. <https://doi.org/10.3109/02699052.2015.1004757>.
(3) Hodgkinson I, Jindrich ML, Metton G, Berard C. Bassin oblique, luxation de hanche et scoliose dans une population de 120 adultes polyhandicapés.Étude descriptive. Annales de Réadaptation et de Médecine Physique 2002;45:57–61. <https://doi.org/10.1016/S0168-6054(01)00177-5>.
(4) de Lattre C, Hodgkinson I, Bérard C. Retentissement de la scoliose chez les patients polyhandicapés: étude descriptive de 61 enfants et adultes polyhandicapés avec ou sans arthrodèse vertébrale. Annales de Réadaptation et de Médecine Physique 2007;50:218–24. <https://doi.org/10.1016/j.annrmp.2007.01.006>.

***To evaluate surgical outcomes, a minimum 2-year follow-up should be used rather than 1-year follow-up.***

- We thank you for your methodological comment on the relevant follow-up duration.
- To the best of our knowledge, this is the first study assessing the clinical severity of PIMD/Polyhandicap adolescents before and after spinal fusion surgeries. The main outcome was clinical, using a validated tool whose responsiveness was accurately defined (5).
- Indeed a 2-year follow-up would have been better, although follow-up at one year avoided too many people being lost and allow to provide quickly these results to medical community. To consider your comment we added in the Strengths and limitations section (p21-22 lines 466-77), the following sentence : “Longer follow-up (2-5 years) would enable us to assess the long-term effect of spinal fusion surgery on the severity health status of patients with PLH, thus further prospective studies with a longer follow-up are needed (therefore, the present one-year follow-up evaluation should not be considered as the final evaluation).”

Reference:

(5) Rousseau M-C, Baumstarck K, Hamouda I, Valkov M, Felce A, Khaldi-Cherif S, et al. Development and initial validation of the polyhandicap severity scale. Revue Neurologique 2020:S0035378720306810. <https://doi.org/10.1016/j.neurol.2020.06.018>.

***As mentioned by the authors, the preoperative assessment was done retrospectively by referring to the medical notes and this will create a major bias.***

- We thank you for discussing the study limitations in this comment.
- Indeed we know there are inherent biases in retrospective cohort methodology, we mentioned them in the Strengths and limitations section (p21-22 lines 466-77).
- We also provided the STROBE checklist following guidelines (6), in supplemental material
- We tried to minimize the risk of bias as much as possible:
  - All the investigators had good training in clinical practice and in research. The protocol and its *a priori* methodology were approved by the institutional review board (Institutional Review Board : IORG0007394 - IRBN492021/CHUSTE – “Terre d’Ethique” Research Committee – Hospital Centre of Saint-Etienne).
  - Even though it is inherent to retrospective methodology: X-rays interpretation were completed by two independent evaluators who were not involved in the surgical procedure. Similarly, preoperative and postoperative data collection from medical record was performed by an independent evaluator not involved in the surgical procedure nor medical post operative follow-up. We added this notion in the Material and Methods - data section (p8, lines 199-201) : “Data extraction was completed from computerized charts by the first author (HB) who was not involved in the surgical procedure nor medical postoperative follow-up. Method for fulfilling evaluation form was as exhaustive as possible to limit risk of bias.”
  - We further discuss the retrospective methodology in the Reviewer #2 – research design comment
  - Loss of information was clearly indicated in the Results - descriptive data section (p13 lines 289 – 291) and in the Fig 5.

Reference:

(6) Vandenbroucke JP, von Elm E, Altman DG, Gøtzsche PC, Mulrow CD, Pocock SJ, et al. Strengthening the Reporting of Observational Studies in Epidemiology (STROBE): explanation and elaboration. PLoS Med 2007;4:e297. <https://doi.org/10.1371/journal.pmed.0040297>.

***The sample population is heterogenous, perhaps should only focus on 1 diagnosis e.g. cerebral palsy.***

- We thank you for the opportunity to make clearer our study. PIMD/polyhandicap meet a variety of etiologies (progressive and non progressive), although the plurality of impairments and induced activity limitations may create a heterogeneous group of patients. To ensure the homogeneity of our population, we used inclusion criteria based on validated tools (GMFCS, FIM). Moreover, these patients presented specific characteristics about scoliosis, mainly severe axial hypotony without spasticity.
- Recently, the Ithaca European Reference Network (ERN) for congenital malformations and rare intellectual disabilities has agreed on the term PIMD/Polyhandicap. An international consensual definition was proposed: “PIMD/Polyhandicap is a complex disability condition occurring in an immature brain, leading to profound intellectual disabilities (IQ<25), sensory impairments and severe motor disabilities, manifesting in the inability to move independently. PIMD/Polyhandicap persons often also have other chronic problems such as nutritional problems, epilepsy, malformations (often in the hips and back) and recurring airway infections. PIMD/Polyhandicap persons need permanent health and educational support, and are dependent on human and technical assistance on a life-long basis” (7).
- We added the corresponding reference in Material and Methods - participants section (p7, line 160) and to our Bibliography [18].

Reference:

(7) Rousseau M-C, Winance M, Baumstarck K. Polyhandicap, profound intellectual multiple disabilities : Concept and definition of a highly specific public health issue. Rev Epidemiol Sante Publique 2023;71:102184. <https://doi.org/10.1016/j.respe.2023.102184>.

***The sample size is low. As highlighted by the authors, a multicenter study should be conducted instead.***

- No data specifically for PIMD/Polyhandicap had been reported prior to our study. The clinical scales PSS and PolyQoL we used have been published respectively in 2021 and 2023, making our article an original work, even if monocentric.
- We are now particularly involved in this issue; actually a French multicentric focus group has been setten up, under the aegis of the French Neuropediatric Society and we hope it will allow to continue our work in a multicenter setting to avoid certain previous methodological biases.

***All patients had complications (100%), and there were 2 deaths out of 30 patients who underwent surgery (6.7% risk of death).***

- Peri and postoperative complications were ranged according to the modified Clavien-Dindo-Sink classification published in 2018 (8), which aims at stratifying accurately surgical complications from minor (grade I) to major complications, and death (grade V). For instance, grade III is defined as a complication that is treatable but requires surgical, endoscopic, or interventional radiology procedure(s), or an unplanned hospital readmission (e.g early neurologic deficit, early skin nonunion, postoperative mechanical failure requiring surgery, deep infection treatable with IV antibiotics, surgical hematoma requiring drainage or debridement, digestive complications, heterotopic ossification requiring surgical excision, late bone nonunion). We detailed the types of complications in Fig 5. and in Results - descriptive data section (p15 lines 308-315).
- We totally agree with your statement. We stated in the Discussion (p18, lines 381-83): “Our hypothesis was confirmed as PSS, coPSS and abPSS scores were significantly improved. We have nevertheless highlighted a 100% rate of complications and 2 deaths, confirming the complexity of these surgeries”. Those rates are in accordance with the literature (9).

References:

(8) Dodwell ER, Pathy R, Widmann RF, Green DW, Scher DM, Blanco JS, et al. Reliability of the Modified Clavien-Dindo-Sink Complication Classification System in Pediatric Orthopaedic Surgery. JBJS OA 2018;3:e0020. <https://doi.org/10.2106/JBJS.OA.18.00020>.
(9) Warner W. Point of View. Journal of the Pediatric Orthopaedic Society of North America 2019;1. <https://doi.org/10.55275/jposna-2019-42>.

***The authors did not mention if there were any neurologic deficits, venous thromboembolism, rate of unplanned return to OR. These are some important complications that are important to spine surgeons.***

***While surgical intervention showed some improvement in the severity health status in this group of patients, one should be aware of the extremely high rate of perioperative complications.***

- To consider this comment, we completed the manuscript as follow, in the Results- descriptive data section (p15 lines 311-12): “neither neurologic, nor digestive or pancreatic complications were noted postoperatively in the patients”.
- To highlight this issue, we also added this sentence in the Conclusion section (p23 lines 489-492): “While spinal fusions showed some improvement in the severity health status, one should be aware of the extremely high rate of perioperative complications fortunately treatable but often requiring readmission, making multidisciplinary expertise mandatory”

**Reviewer #2**

***The study investigates the impact of spinal fusion surgeries on the severity of health status in adolescents with polyhandicap and scoliosis. It is a monocentric retrospective observational study, examining patients who underwent spinal fusion surgeries at the University Hospital Centre of Saint-Etienne, France, from 2009 to 2020. The primary outcome measured was the variation in the Polyhandicap Severity Scale (PSS) score post-surgery, with secondary outcomes including variations in PSS subscores, quality of life scores, X-ray parameters, and surgical complication rates and lengths of stay.***

***Data for the study were collected using a specific research algorithm and independently reviewed. Variables of interest were extracted from eligible charts, and families were contacted to document their perspectives on the adolescents' quality of life post-surgery. Statistical analysis included checking for normal distribution of variables, using paired t-tests, Wilcoxon matched-pairs signed rank tests, and univariate analysis with Mann-Whitney tests and Spearman correlation matrices.***

***The study included 30 patients (19 women and 11 men) who met the definition of polyhandicap. Significant improvements were observed between pre- and postoperative PSS scores, particularly in aspects like pain, respiratory, digestive, and skin disabilities. However, there was a 100% rate of complications, with at least one complication per patient and a mortality rate of 6.7% during follow-up.***

***While the study offers valuable insights into the effects of spinal fusion on adolescents with polyhandicap, addressing these points could enhance the depth and applicability of the findings:***

***1. Research design: The retrospective observational nature of the study is appropriate for the research question, but the existence of confounding factors, such as patient demographic information, consistency of the surgery, post-surgery treatments etc., limits the generalizability of the findings. Providing information or comment on this would strengthen the study's credibility and validity.***

- We thank you for your comment. Patient demographic information are provided in Table 1. and Results - descriptive data section
- We added all the surgical protocol information’s in Material and methods – participants section (p7-8 lines 164-179):

“Surgery consisted in all patients in an hybrid instrumentation of the spine: patients in supine position; pelvic fixation to correct the pelvis obliquity; instrumentation of the lumbar area with pedicular screws; thoracic area with sublaminar ligaments and hook-claw at the upper part of the instrumentation; rods were self-bowing by surgeon during the procedure; all implants from Medtronic (Medicrea™, Rillieux la Pape 69140, France).

Postoperative care systematically included one night in ICU for monitoring. Pain medication started peroperatively with intrathecal injection of morphine (5 µg/kg). A peridural catheter was maintained during 48h to 5 days with continual perfusion of low dose ropivacaine 0.2% (related to patient weight), and if necessary, paracetamol, ketoprofen, diazepam and oral morphine (related to patient weight and respiratory status) could be used. Esomeprazole for gastric ulcer prevention, lactulose per oral for constipation prevention, and enoxaparin if necessary (related to risk factors) were also administered. Feeding started one day after spinal fusion, through various modalities depending on patients. Lower limbs mobilizations were started two days postoperatively in sitting position, then performed every day.”

***2. Statistical analysis: While the authors provide mean and standard deviations, the significance levels of the analyses should be clearly indicated. Additionally, a graphical representation of each patient's pre- and post-surgery scores would provide a clearer understanding of the data distribution, highlighting any potential skewness or outliers.***

- We thank you for your meticulous analysis and for this opportunity to better present our results
  - Authors guidelines mentioned p-values less than 0.001 may be expressed as p < 0.001. We nevertheless added accurate significance levels in Table 2. in the dedicated p-values column.
  - We formatted an additional pre/post chart of each patient’s pre and postoperative PSS scores to help in better data visualization. See “Fig 4. Scatter plot of individual preoperative versus postoperative PSS scores.”

**Fig 4. Scatter plot of individual preoperative versus postoperative PSS scores**. Mean PSS evolution with standard error is represented by the large red solid line with error bar. PSS evolution *i.e* improvement (n=18; dark solid line), stability (n=8; grey solid line), worsening (n=1; grey dashed line) are represented for each individual.

***Implications and limitations of the study: I suggest the authors mentioning the practical implications and limitations of the study, which is important for understanding the scope and applicability of the findings.***

- To consider this comment, we completed the Strengths and limitations section (p22 lines 466-77) as follows: “One of the main issues when performing spinal fusions in patients with PLH is the assessment of predicted benefits. This study aimed to support the rationale for spinal fusion, acknowledging the already known complication rates and risks in PLH adolescents. Considering the severity of the health status evaluation of these patients and the expected improvement of the global health status and comfort after spine surgery for scoliosis, each case requires careful consideration and evaluation. We support that, under the condition of acceptable preoperative health status, the indication for spinal fusion in that population could be relevant and reasonable. While retrospective evaluation is questionable, the rigorous method used during data collection should limit this bias. The PolyQoL questionnaire has been supported by the verbatim collected in a part of the population, and obviously the good health status of the patients at final follow-up confirms our hypothesis.”
